# *Dracocephalum heterophyllum* (DH) Exhibits Potent Anti-Proliferative Effects on Autoreactive CD4^+^ T Cells and Ameliorates the Development of Experimental Autoimmune Uveitis

**DOI:** 10.3389/fimmu.2020.575669

**Published:** 2020-10-08

**Authors:** Jiang Bian, Ke Wang, Qilan Wang, Pu Wang, Ting Wang, Weiyun Shi, Qingguo Ruan

**Affiliations:** ^1^Qingdao Eye Hospital of Shandong First Medical University, Qingdao, China; ^2^State Key Laboratory Cultivation Base, Shandong Provincial Key Laboratory of Ophthalmology, Shandong Eye Institute, Shandong First Medical University & Shandong Academy of Medical Sciences, Qingdao, China; ^3^Department of Ophthalmology, Qingdao University Medical College, Qingdao, China; ^4^Northwest Plateau Institutes of Biology, Chinese Academy of Sciences, Xining, China; ^5^Center for Antibody Drug, Institute of Biomedicine and Biotechnology, Shenzhen Institutes of Advanced Technology, Chinese Academy of Sciences, Shenzhen, China

**Keywords:** autoimmunity, proliferation, herbal medicine, T cell, experimental autoimmune uveitis

## Abstract

Experimental autoimmune uveitis (EAU) is a CD4^+^ T cell–mediated organ-specific autoimmune disease and has been considered as a model of human autoimmune uveitis. *Dracocephalum heterophyllum* (DH) is a Chinese herbal medicine used in treating hepatitis. DH suppressed the production of inflammatory cytokines through the recruitment of myeloid-derived suppressor cells (MDSCs) to the liver. However, it remains elusive whether DH can directly regulate CD4^+^ T cell biology and hence ameliorates the development of CD4^+^ T cell–mediated autoimmune disease. In the current study, we found that DH extract significantly suppressed the production of pro-inflammatory cytokines by CD4^+^ T cells. Further study showed that DH didn’t affect the activation, differentiation, and apoptosis of CD4^+^ T cells. Instead, it significantly suppressed the proliferation of conventional CD4^+^ T cells both *in vitro* and *in vivo*. Mechanistic study showed that DH-treated CD4^+^ T cells were partially arrested at the G2/M phase of the cell cycle because of the enhanced inhibitory phosphorylation of Cdc2 (Tyr15). In addition, we demonstrated that treatment with DH significantly ameliorated EAU in mice through suppressing the proliferation of autoreactive antigen specific CD4^+^ T cells. Taken together, the current study indicates that DH-mediated suppression of CD4^+^ T cell proliferation may provide a promising therapeutic strategy for treating CD4^+^ T cell–mediated diseases.

## Introduction

Experimental autoimmune uveitis (EAU) is an animal disease model of human posterior autoimmune uveitis and can be induced in susceptible animals by immunization with retinal antigens (1). EAU resembles the key immunological characteristics of autoimmune uveitis in humans, as both are CD4^+^ T cell–mediated (2–4). The proliferation and differentiation of autoreactive retinal antigen-specific CD4^+^ T cells is the major cause of the initiation and progression of EAU (5, 6). Among different types of CD4^+^ T cells, IFN-γ-producing Th1 cells and IL-17–producing Th17 cells were implicated as the major contributors to the progression of EAU (7). After infiltrating into the retina, they recruit inflammatory cells such as macrophages and monocytes, which produce TNF-α and other pro-inflammatory cytokines that lead to the damage of retina tissue. (8–10). Since T cells are central regulators of the immune response, it is essential to maintain a normal T cell homeostasis, as its deregulation can lead to immunodeficiency or autoimmunity (11–13).

*Dracocephalum heterophyllum* (DH) is a traditional Chinese medicine possesses various pharmacological effects including anti-inflammatory, antimicrobial and antioxidant activities (14). There have been reports showing that this herb can be used to treat human disorders such as hypertension, cardiomyocyte hypertrophy, cough, bronchitis and hepatitis (15, 16). However, the pharmacological mechanism by which DH-mediated protective effects in hepatitis has only recently been revealed. Zheng et al. reported that DH extract significantly ameliorated liver injury and suppressed the production of inflammatory cytokines by T cells through recruiting CD11b^+^Gr1^+^ myeloid derived suppressor cells (MDSCs) to the liver (16). However, it remains elusive whether DH is capable of directly regulating CD4^+^ T cell biology including activation, differentiation, apoptosis, and proliferation.

In the current study, we evaluated the direct effects of DH on CD4^+^ T cells. Our study showed that DH didn’t affect the apoptosis, activation and differentiation of CD4^+^ T cells. Instead, it suppressed the production of inflammatory cytokines by conventional CD4^+^ T cells through the inhibition of their proliferation. Mechanistic study revealed that DH-treated CD4^+^ T cells were partially arrested at the G2/M phase of the cell cycle because of the enhanced inhibitory phosphorylation of Cdc2 (Tyr15). Furthermore, we demonstrated that treatment with DH significantly ameliorated EAU in mice through suppressing the proliferation of autoreactive retinal antigen-specific CD4^+^ T cells.

## Materials and Methods

### Animals

6 to 8-week old female mice in the C57BL/6 background were purchased from Beijing Vital River Laboratory Animal Technology Company Limited. 6 to 8-week old Foxp3-YFP transgenic mice were kindly provided by Dr. Bin Li from Shanghai Jiao Tong University, P. R. China. Mice were kept under pathogen-free conditions at the animal core facility of Shandong Eye Institute, Shandong First Medical University & Shandong Academy of Medical Sciences. All efforts were made to minimize the number of mice used and to less animal distress, pain, and injury. All experiments were carried out in accordance with the Committee guidelines of Shandong Eye Institute and the Association for Research in Vision and Ophthalmology (ARVO) Statement for the Use of Animals in Ophthalmic and Vision Research.

### Preparation of DH Extract

The medicinal DH was collected and pulverized into powder by a mechanical grinder. The powder was then macerated in 95% ethanol and filtered to remove the residue. The filtered extract was concentrated in a rotary evaporator at 40°C, followed by removing ethanol and water using freeze drier. The ethanol extract was further dispersed in water and then extracted with ethyl acetate to obtain the ethyl acetate fraction. The ethyl acetate fraction (5.0 mg/ml) was analyzed by HPLC (Column: Odyssil C18 (250 mm × 4.6 mm, 5 μm); Mobile phase: (A) 0.2% formic acid in water, (B) methanol; Gradient elution: time 0 at 20% B to 60 min at 100% B; Injection volume: 10 μl; Detection wavelength: 254 nm). To prepare DH extract for experiments, the ethyl acetate extract was first dissolved in dimethyl sulfoxide (DMSO) and then diluted to working concentrations with cell culture medium or PBS buffer. Two different batches of the DH extract were used in this study and no batch to batch variation of the preparation was found regarding the key data obtained.

### Induction and Clinical Evaluation of EAU

6- to 8-week-old C57BL/6 mice were anesthetized by intraperitoneal injection of pentobarbital sodium (80 mg/kg). EAU was induced by active immunization as previously described (17). Briefly, mice were immunized with 400 μg inter-photoreceptor retinoid-binding protein (IRBP)1-20 (5 mg/ml, GPTHLFQPSLVLDMAKVLLD, purchased from China Peptides) emulsified 1:1 in complete Freund’s adjuvant (Chondrex) with an additional 100 μl mycobacterium tuberculosis H37R (5 mg/ml, BD biosciences) at six locations (footpads (2×), tail base, posterior neck and bilateral flanks (2×)). Then an additional 200 ng bordetella pertussis toxin (Millipore) was intravenously injected immediately. Starting from day 7 to day 15, mice were systemically administrated (through tail vain injection) with equal volumes (100 μl) of DMSO or 20 mg/kg of DH every 48 h. Mice were sacrificed at the 21st day after immunization and histopathological profiles of the eye were determined by hematoxylin and eosin staining. The severity of EAU was evaluated in a masked fashion on a scale of 0 to 4 using previously published criteria based on the number, type, and size of lesions (18).

### Flow Cytometry Analysis

For *in vitro* experiments, total CD4^+^ T cells were isolated from the mesenteric lymph node (MLN) of naïve 6- to 8-week-old C57BL/6 mice using EasySep™ Mouse CD4^+^ T Cell Isolation Kit (STEMCELL). MLN was used for the isolation of CD4^+^ T cells due to its relatively large size and abundance of T cells. For *in vivo* experiments, single-cell suspension from the eye and draining lymph node (cervical lymph node, CLN) were collected from normal and EAU mice (21 days after immunization) as previously described (6, 19). Briefly, eyes were enucleated and minced followed by removing lens, digested for 10 min at 37°C with collagenase D (1 mg/ml, Roche) and DNase I (100 μg/ml, Roche) in complete RPMI-1640 medium (Corning). Digested tissues were filtered through 70 μm nylon mesh. Cells were labeled with a combination of the following fluorescence-conjugated mouse mAbs: FITC-anti-CD4, PE-anti-CD4, APC-anti-CD69, APC-anti-CD62L, APC-anti-CD44, Perp-Cy5.5-anti-CD25, FITC-anti-Gr-1, and APC-anti-CD11b. All antibodies were purchased from BioLegend. For the intracellular staining of IL-17A, IFN-γ, IL-4, and Foxp3, naïve CD4^+^ T cells were isolated from the MLN of naïve 6- to 8-week-old C57BL/6 mice using EasySep™ Mouse naïve CD4^+^ T Cell Isolation Kit (STEMCELL) and cultured under conditions that induced Th1, Th2, Th17, or regulatory T (Treg) cells as previously described (20, 21). Cells were then stimulated with cell stimulation cocktail (PMA+Ionomycin) plus protein transport inhibitors (eBioscience) for 4 h. Cell surface staining was first performed with FITC-anti-CD4. After fixation and permeabilization, cells were stained with either PE-anti-IFN-γ, APC-anti-IL-4, APC-anti-IL-17A, or Alexa Flour 647-anti-Foxp3 (BioLegend) per manufacturer’s instructions (Invitrogen). Stained cells were then examined on CytoFLEX flow cytometry system (Beckman Coulter Inc).

### MTS Assay

The MTS assay was performed according to manufacturer’s instructions (Promega). Briefly, total CD4^+^ T cells were isolated from the MLN of naïve 6- to 8-week-old C57BL/6 mice using EasySep™ Mouse CD4^+^ T Cell Isolation Kit. Cells were then cultured in 96-well plate at 2×10^5^ cells/well and stimulated with anti-CD3 (1 μg/ml) plus anti-CD28 (1 μg/ml) in the absence (DMSO control) or presence of DH (10, 20, and 40 mg/L). After 48 h, 20 μl of MTS One Solution Reagent was added into each well and incubate at 37°C for 2 h. The absorbance was determined at 490 nm using a 96-well plate reader (Thermo).

### Preparation of Bone Marrow-Derived Dendritic Cells and Bone Marrow-Derived Macrophages

Bone marrow-derived dendritic cells (BMDCs) and bone marrow-derived macrophages (BMDMs) were generated from femoral and tibial bones cells as previously described (22). For BMDCs, 8×10^6^ bone marrow precursors/ml from C57BL/6 mice were seeded in complete RPMI-1640 culture medium supplemented with 20 ng/ml GM-CSF (PeproTech) and 10 ng/ml IL-4 (PeproTech) in 10 cm cell dish. Half of the medium was replaced with fresh medium on days 3 and 5. On day 6, BMDCs were about 85% CD11c^+^ (BioLegend) as determined by flow cytometry and ready for further experiments. For BMDMs, 8×10^6^ bone marrow precursors/ml from C57BL/6 mice were seeded in complete DMEM culture medium (Corning) supplemented with 20% L929 cell supernatant in 10 cm cell dish. The above-mentioned medium was supplemented with 10 ml fresh medium on day 4. On day 7, BMDMs were about 95% F4/80^+^ (BioLegend) as determined by flow cytometry and ready for further experiments.

### Enzyme-Linked Immunosorbent Assay

For cytokine assays, total CD4^+^ T cells were isolated from the MLN of naïve 6- to 8-week-old C57BL/6 mice using EasySep™ Mouse CD4^+^ T Cell Isolation Kit. The purity of the isolated CD4^+^ T cells is ~ 90%. CD4^+^ T cells were cultured at 2×10^5^ cells/well (96-well plate) in 200 μl complete RPMI-1640 supplemented with 10% FBS (Gibco), 4 mM L-glutamine (Gibco), 1 mM sodium pyruvate (Gibco), 55 µM 2-mercaptoethanol (Sigma), 10 mM HEPES (Gibco), 100 units/ml penicillin and 100 µg/ml streptomycin (Gibco). Cells were stimulated with anti-CD3 (1 μg/ml, eBioscience) plus anti-CD28 (1 μg/ml, eBioscience) in the absence (DMSO control) or presence of DH (10, 20, and 40 mg/L) for 48 h. The concentration of IFN-γ and IL-17A in the cell culture supernatant was determined by Enzyme-linked Immunosorbent Assay (ELISA) per manufacturer’s instructions (eBioscience). Alternatively, total CD4^+^ T cells were isolated from the CLN of IRBP-immunized mice 10 days after immunization. BMDCs were prepared from naïve C57BL/6 mice as mentioned above and treated with LPS (100 ng/ml) for 24 h. Matured BMDCs were furthered purified using EasySep™ Mouse CD11c Positive Selection Kit (STEMCELL). 3×10^5^ CD4^+^ T cells were mixed with 0.6 ×10^5^ matured BMDCs in 96-well plate and stimulated with IRBP peptide (30 μg/ml, China Peptides) in the absence (DMSO control) or presence of DH (10, 20, and 40 mg/L) for 48 h. The concentration of IFN-γ and IL-17A in the cell culture supernatant was determined by ELISA. For the detection of cytokines produced by BMDC and BMDM, cells were stimulated with LPS (1 μg/ml, Sigma-Aldrich) in the absence (DMSO control) or presence of DH (10, 20, and 40 mg/L) for 24 h. The concentration of IL-6 and IL-1β in the cell culture supernatant was determined by ELISA as described above. For the detection of IL-10 production by regulatory T cells (Tregs), CD4^+^ T cells were first enriched from the MLN of Foxp3-YFP transgenic mice using EasySep™ Mouse CD4^+^ T Cell Isolation Kit (STEMCELL). Enriched CD4^+^ T cells were stained with PE-anti-CD4 and Perp-cy5.5-anti-CD25 and CD4^+^CD25^+^YFP^+^ Tregs were sorted using BD FACSAria cell sorter (BD Biosciences, USA). Sorted Tregs were stimulated with anti-CD3 (1 μg/ml) plus anti-CD28 (1 μg/ml) in the absence (DMSO control) or presence of DH (10, 20, and 40 mg/L) for 48 h. The concentration of IL-10 in the cell culture supernatant was determined by ELISA.

### CFSE-Based Cell Proliferation Assay

Total CD4^+^ T cells were isolated from the MLN of naïve 6- to 8-week-old C57BL/6 mice and incubated with 5 μM 5, 6-carboxyfluorescein diacetate succinimidyl ester (CFSE, eBioscience) at 37°C for 15 min. The reaction was stopped with the same volume of cold RPMI-1640 containing 10% heat-inactivated FBS. After washing three times with PBS, CFSE-labeled cells were cultured at 2×10^5^ cells/well (96-well plate) and stimulated with anti-CD3 (1 μg/ml) plus anti-CD28 (1 μg/ml) in the absence (DMSO control) or presence of DH (10, 20, and 40 mg/L) for 72 h. Cell proliferation was then analyzed by flow cytometry. Un-stimulated cells were used as negative control.

### *In Vitro* BrdU Assay

Total CD4^+^ T cells isolated from the CLN of IRBP-immunized mice (10 days after immunization) were cultured with 10 μM BrdU (BD Bioscience) at 3×10^5^ cells/well (96-well plate) and stimulated with IRBP-loaded BMDC (0.6×10^5^ cells) in the absence (DMSO control) or presence of DH (10, 20, and 40 mg/L) for 72 h. After surface staining with FITC-anti-CD4, cells were stained with APC-anti-BrdU according to manufacturer’s instructions (BD Bioscience) and analyzed by flow cytometry.

### *In Vivo* BrdU Assay

Mice were first intravenously injected with DMSO or DH (20 mg/kg). After 12 h, mice were intraperitoneally injected with 100 µl of 10 mg/ml BrdU. Administration of BrdU was repeated every 12 h for three consecutive days. 12 h after the last treatment, Total cells were isolated from Peyer’s patch (PP), intestinal epithelial cell (iEC) and lamina propria (LP). Cell surface staining was first performed with FITC-anti-CD4. Cells were then stained with APC-anti-BrdU according to manufacturer’s instructions (BD Bioscience) and analyzed by flow cytometry.

### Cell Cycle Analysis

Propidium iodide (PI) staining was used to analyze DNA contents and cell cycle. Briefly, total CD4^+^ T cells were isolated from the MLN of naïve 6- to 8-week-old C57BL/6 mice using EasySep™ Mouse CD4^+^ T Cell Isolation Kit. Cells were cultured in 96-well plate at 2×10^5^ cells/well and stimulated with anti-CD3 (1 μg/ml) plus anti-CD28 (1 μg/ml) in the absence (DMSO control) or presence of 40 mg/L DH for 24 h or 48 h. Cells neither stimulated nor treated with DH were used as control (0 h). Alternatively, total CD4^+^ T cells were isolated from the CLN of IRBP-immunized mice (10 days after immunization) and stimulated with IRBP-loaded BMDCs in the absence (DMSO control) or presence of DH (10, 20, and 40 mg/L) for 48 h. After surface staining with FITC-anti-CD4 antibody, cells were fixed and incubated with staining buffer containing 50 µg/ml PI and 0.25 mg/ml RNase A (Beyotime) at room temperature for 30 min. Cell cycle distribution was determined using flow cytometer analysis (Beckman Coulter). The percentages of cells in the G0/G1, S, and G2/M phases were determined using Beckman software.

### Annexin V/PI Assay

The percentage of live cells was determined by Annexin V/PI (Propidium Iodide) staining and flow cytometry analysis according to manufacturer’s instructions (KeyGEN BioTECH). Briefly, cells were firstly stained with FITC-anti-CD4 and then stained with APC-anti-Annexin V and PI followed by analysis using flow cytometry (Beckman Coulter Inc). Live cells were defined as Annexin V and PI double-negative cells.

### Western Blot Analysis

Total CD4^+^ T cells were isolated from the MLN of naïve 6- to 8-week-old C57BL/6 mice using EasySep™ Mouse CD4^+^ T Cell Isolation Kit. Cells were then cultured in 24-well plate at 5×10^6^ cells/well and stimulated with anti-CD3 (1 μg/ml) plus anti-CD28 (1 μg/ml) in the absence (DMSO control) or presence of 40 mg/L DH. Alternatively, total CD4^+^ T cells were isolated from the CLN of IRBP-immunized mice (10 days after immunization) and stimulated with IRBP-loaded BMDCs in the absence (DMSO control) or presence of DH (10, 20, and 40 mg/L). After 24 h or 48 h, cells were collected and treated with RIPA lysis buffer (Beyotime) and protein concentration was determined using the Pierce BCA protein assay kit (Beyotime). Equal amounts of protein were loaded and subjected to sodium dodecyl sulfate-polyacrylamide gel electrophoresis (SDS-PAGE) and then transferred to PVDF membranes (Millipore) using BIO-RAD Mini Trans-blot. Membranes were blocked with 5% BSA and then incubated overnight at 4°C with rabbit anti-p-Cdc2 (CST) and rabbit anti-Wee1 (Abcam), followed by incubation with HRP-conjugated secondary antibody. GAPDH was used as loading control. Protein bands were visualized using chemiluminescence imaging system (BIO-RAD).

### Statistical Analysis

The data were analyzed using GraphPad Prism 8.0 and expressed as mean ± SD. The student’s *t*-test was used to compare the significance of the difference between two groups. ANOVA was utilized for multi-group comparison, with Tukey’s test for subsequent post-hoc comparisons between two groups. Differences were considered statistically significant at *P<0.05, **P<0.01, and ***P<0.001.

## Results

### DH Suppresses the Production of Pro-Inflammatory Cytokines by Polyclonally Activated CD4^+^ T Cells

**Figure 1:**
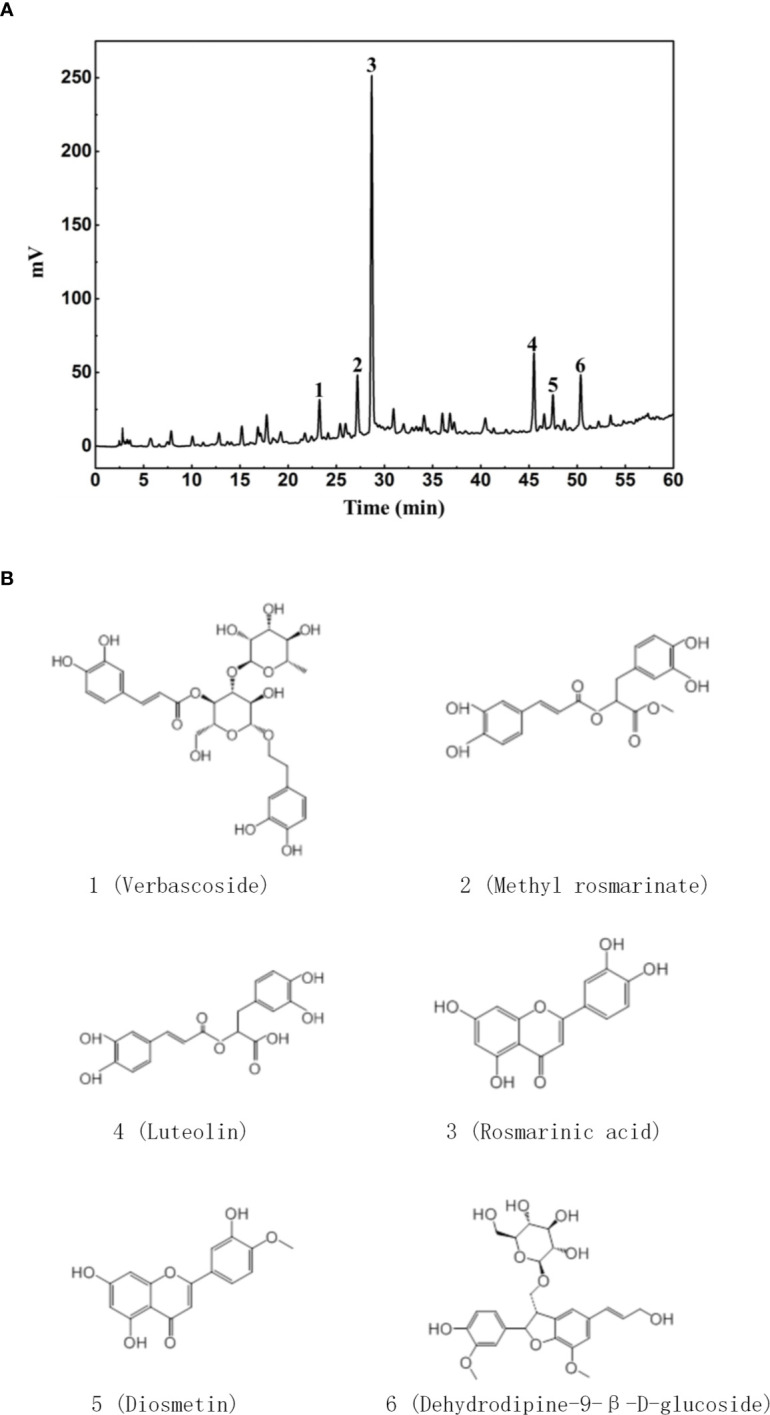
Characterization of DH ethyl acetate fraction. DH ethyl acetate fraction was prepared as described in the materials & methods. The main components **(A)** in the fraction and their chemical formula **(B)** were determined by HPLC.

**Figure 2:**
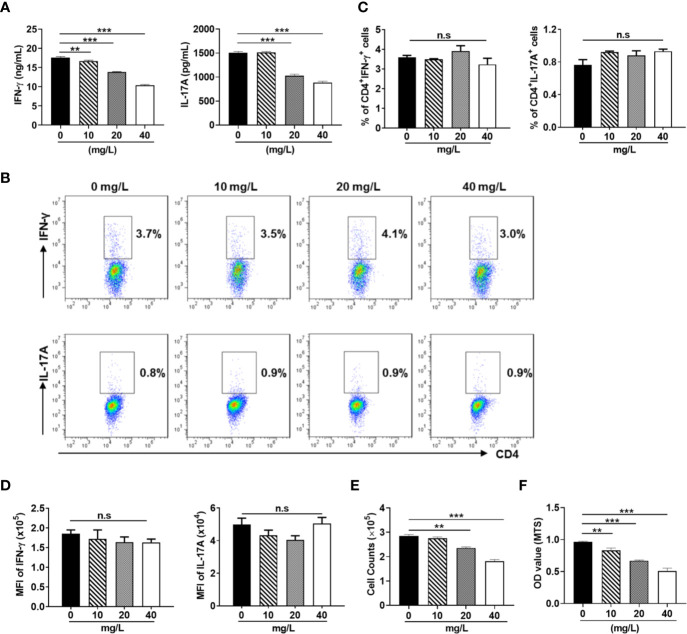
DH suppresses the production of inflammatory cytokines by CD4^+^ T cells. CD4^+^ T cells were isolated from the mesenteric lymph node (MLN) of naïve 6-8-week-old C57BL/6 mice. Cells were cultured in 96-well plate at 2×10^5^ cells/well and stimulated with anti-CD3 (1 μg/ml) plus anti-CD28 (1 μg/ml) in the absence (0 mg/L, DMSO control) or presence of DH (10, 20, and 40 mg/L). After 48 h, **(A)** culture supernatants were collected and the concentration of IFN-γ and IL-17A was determined by ELISA. **(B–D)** cells were re-stimulated with PMA plus ionomycin in the presence of protein transport inhibitor for 4 h. Cell surface staining was first performed with FITC-anti-CD4. Cells were then fixed, permeated, stained with PE-anti-IFN-γ and APC-anti-IL-17A, and analyzed by flow cytometry. The dot plot **(B)** and quantification **(C)** of the percentages of CD4^+^IFN-γ^+^ and CD4^+^IL-17A^+^ cells, as well as quantification of the mean fluorescence intensity (MFI) of IFN-γ and IL-17A **(D)** were shown. Cells shown in dot plots were gated on CD4^+^ T cells. **(E)** the number of CD4^+^ T cells was counted. **(F)** MTS assay was performed according to manufacturer’s instructions. Data shown are mean±SD **(C, D)** or representative (all other panels) of three independent experiments. n.s., no significance. **P < 0.01, ***P < 0.001.

DH was first extracted with ethanol and then ethyl acetate. Since previous study has revealed that ethyl acetate extract showed superior anti-inflammatory effect than N-butanol extract and petroleum ether extract (14), DH ethyl acetate extract was used in this study. HPLC analysis revealed that DH ethyl acetate extract (hereinafter referred to as DH) is composed of six main components (**Figure 1A**), which includes Verbascoside, Methyl rosmarinate, Rosmarinic acid, Luteolin, Diosmetin, and Dehydrodipine-9-β-d-glucoside (**Figure 1B**). To determine the direct effect of DH on cytokine production by CD4^+^ T cells, we isolated total CD4^+^ T cells from the MLN of naïve C57BL/6 mice. Cells were then stimulated *in vitro* with anti-CD3 plus anti-CD28 and treated with or without DH. Our results showed that DH significantly decreased the production of IFN-γ and IL-17A by CD4^+^ T cells (**Figure 2A**). The decreased production of cytokines could be due to the decreased ability of CD4^+^ T cells to produce cytokines or reduced number of CD4^+^ T cells upon DH treatment. We then examined the per-cell-based production of cytokines by CD4^+^ T cells using flow cytometry analysis. When gated on CD4^+^ T cells, our results showed that neither the percentage (**Figures 2B, C**) nor the mean fluorescence intensity (MFI) (**Figure 2D**) of IFN-γ^+^ and IL-17A^+^ cells was affected upon DH treatment.

We next examined whether the number CD4^+^ T cells was affected upon DH treatment. Our results showed that DH significantly reduced CD4^+^ T-cell number at the end of cell culture (**Figure 2E**). This result was further confirmed by MTS assay (**Figure 2F**), which allows assessing both the viability and proliferation of cells.

**Figure 3:**
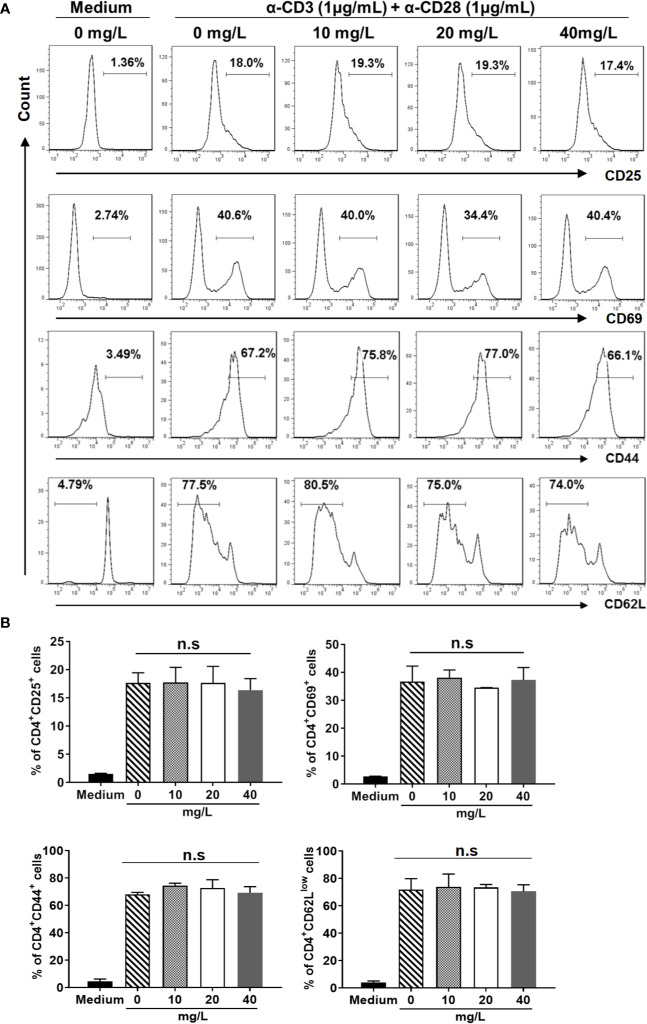
DH doesn’t affect CD4^+^ T-cell activation *in vitro*. Naïve CD4^+^ T cells (for the detection of CD25) and total CD4^+^ T cells (for the detection of CD44, CD62L and CD69) were isolated from the mesenteric lymph node (MLN) of naïve 6-8-week-old C57BL/6 mice. Cells were then cultured in 96-well plate at 2×10^5^ cells/well and stimulated with anti-CD3 (1 μg/ml) plus anti-CD28 (1 μg/ml) in the absence (0 mg/L, DMSO control) or presence of DH (10, 20, and 40 mg/L). Cells neither stimulated nor treated with DH were used as negative control (Medium). After 6 h (for CD25 and CD69) or 48 h (for CD44 and CD62L), cells were stained with FITC-anti-CD4 plus Perp-Cy5.5–anti-CD25, APC-anti-CD69, APC-anti-CD44 or APC-anti-CD62L, and analyzed by flow cytometry. The histogram **(A)** and quantification **(B)** of the percentages of CD4^+^CD25^+^, CD4^+^CD69^+^, CD4^+^CD44^+^, and CD4^+^CD62L^low^ cells were shown. Cells shown in histogram plots were gated on CD4^+^ T cells. Data are representative **(A)** or shown as mean±SD **(B)** of two independent experiments. n.s., no significance.

**Figure 4:**
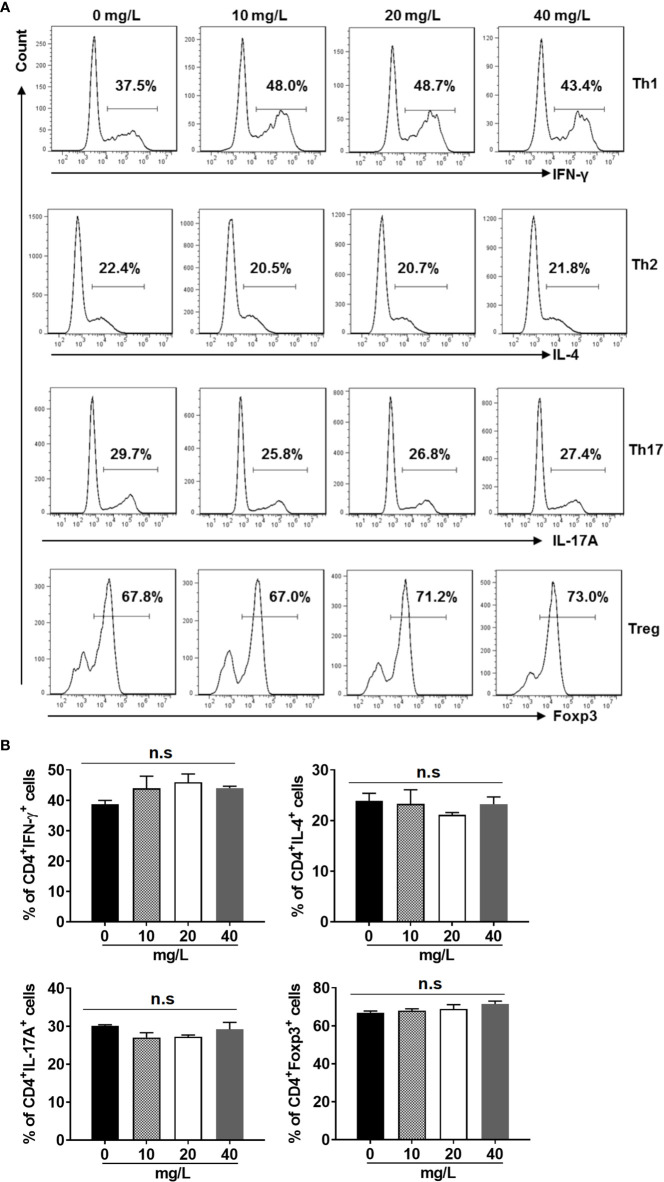
DH doesn’t affect CD4^+^ T-cell differentiation *in vitro*. Naїve CD4^+^ T cells were isolated from the mesenteric lymph node (MLN) of naïve 6-8-week-old C57BL/6 mice. Cells were then cultured in 96-well plate at 2×10^5^ cells/well under Th1, Th2, Th17 or Treg-inducing conditions and treated in the absence (0 mg/L, DMSO control) or presence of DH (10, 20, and 40 mg/L). After 72 h, cell surface staining was first performed with FITC-anti-CD4. Cells were then fixed, permeated, and stained with PE-anti-IFN-γ, APC-anti-IL-4, APC-anti-IL-17A or Alexa Flour 647-anti-Foxp3, respectively. The histogram **(A)** and quantification **(B)** of the percentages of CD4^+^IFN-γ^+^, CD4^+^IL-4^+^, CD4^+^IL-17A^+^ and CD4^+^Foxp3^+^ cells were shown. Cells shown in histogram plots were gated on CD4^+^ T cells. Data are representative **(A)** or shown as mean±SD **(B)** of three independent experiments. n.s, no significance.

In addition, we examined the activation (**Figures 3A, B**) and differentiation (**Figures 4A, B**) of CD4^+^ T cells treated with or without DH. Our results showed that both were comparable between DH treated and DMSO control group.

### DH Exhibits Anti-Proliferative Effects on Polyclonally Activated CD4^+^ T cells

**Figure 5:**
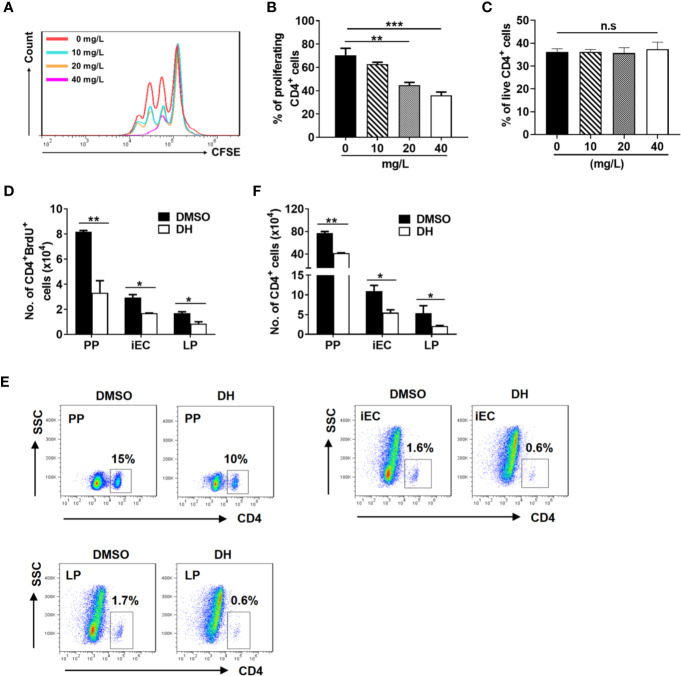
DH suppresses the proliferation of CD4^+^ T cells both *in vitro* and *in vivo*. **(A, B)** CD4^+^ T cells were isolated from the MLN of naïve 6-8-week-old C57BL/6 mice and labeled with CFSE. Cells were then cultured in 96-well plate at 2×10^5^ cells/well and stimulated with anti-CD3 (1 μg/ml) plus anti-CD28 (1 μg/ml) in the absence (0 mg/L, DMSO control) or presence of DH (10, 20, and 40 mg/L). After 72 h, cell proliferation was examined by flow cytometry. The histogram **(A)** and quantification **(B)** of the proliferating CD4^+^ T cells were shown. Proliferating cells were defined as those showed at least one-folder dilution of CFSE. **(C)** CD4^+^ T cells were isolated and cultured as in **(A, B)** but without labeling with CFSE. After 48 h, cells were stained with APC-Annexin-V and PI, and then analyzed by flow cytometry. Data are presented as the percentage of live cells (Annexin V^-^PI^-^). **(D–F)** 6-8-week-old C57BL/6 mice were first intravenously injected with DMSO or DH (20 mg/kg). After 12 h, mice were intraperitoneally injected with 100 μl of 10 mg/ml BrdU. Administration of BrdU was repeated every 12 h for three consecutive days. 12 h after the last treatment, mice were sacrificed and total cells were isolated from PP, iEC, and LP. Cell surface staining was first performed with FITC-anti-CD4. Cells were then stained with APC-anti-BrdU and the total number of CD4^+^BrdU^+^ cells **(D)**, the percentage **(E)** and total number **(F)** of CD4^+^ T cells were determined by flow cytometry. Cells shown in dot plots were gated on total live cells. Data are representative **(A, E)** or shown as the mean±SD (all other panels) of two independent experiments. PP, Peyer’s patches; iEC, intestinal epithelial cells; LP, lamina propria cells. n.s, no significance. *P < 0.05, **P < 0.01, ***P < 0.001.

Reduced CD4^+^ T-cell number upon DH treatment could be due to decreased cell proliferation and/or increased cell death. We then first examined the proliferation of anti-CD3 plus anti-CD28 stimulated CD4^+^ T cells using CFSE-based cell proliferation assay. Our results showed that DH treatment significantly suppressed the proliferation of CD4^+^ T cells (**Figures 5A, B**). Next, we examined the degree of cell death using Annexin-V/PI staining. Our results showed that, after stimulation with anti-CD3 plus anti-CD28 for 48 h, the percentage of live cells (Annexin-V^-^PI^-^) (**Figure 5C**) was comparable between DH treated and DMSO control group.

In addition, we used labeling with the thymidine analog BrdU to assess the effect of DH on CD4^+^ T cell proliferation *in vivo*. Our results showed that the number of BrdU-positive CD4^+^ T cells from Peyer’s patches (PP), intestinal epithelial cells (iEC) and lamina propria cells (LP) was greatly decreased when mice were treated with DH (**Figure 5D**). In line with this, the percentage (**Figure 5E**) and number (**Figure 5F**) of CD4^+^ T cells were also decreased in the PP, iEC, and LP from DH-treated mice.

**Figure 6:**
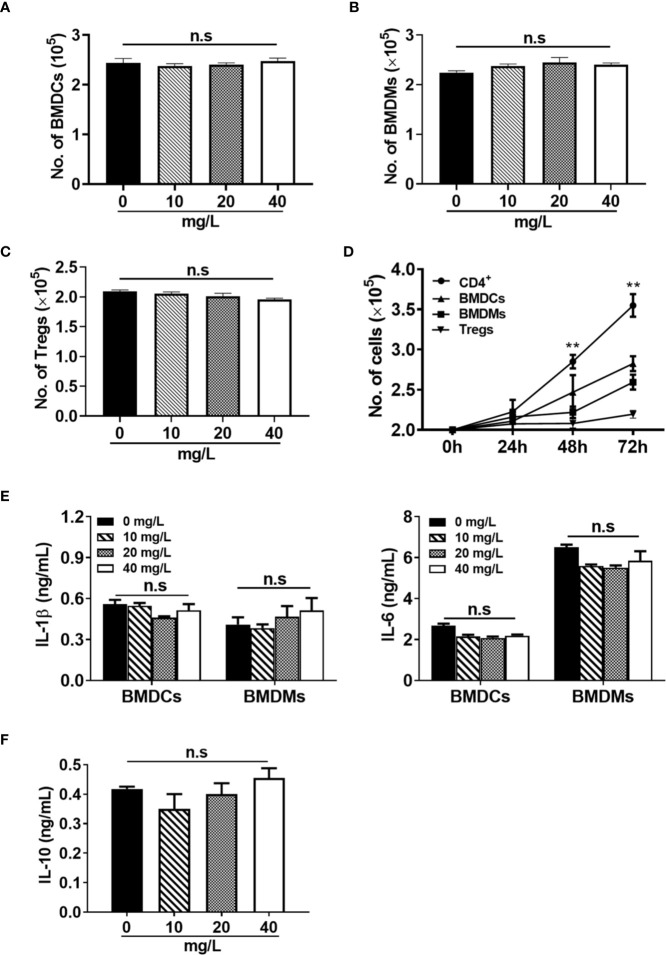
DH does not affect the proliferation of myeloid cells and regulatory T cells. **(A, B)** BMDCs **(A)** and BMDMs **(B)** were cultured in 96-well plate at 2×10^5^ cells/well and treated with or without different concentrations of DH as indicated. Total cell number was counted 48 h after treatment. **(C)** CD4^+^CD25^+^YFP^+^ Tregs were isolated from Foxp3-YFP transgenic mice and cultured in 96-well plate at 2×10^5^ cells/well. Cells were treated with anti-CD3 (1 μg/ml) plus anti-CD28 (1 μg/ml) in the absence (0 mg/L, DMSO control) or presence of different concentrations of DH as indicated. Total cell number was counted 48 h after treatment. **(D)** BMDCs, BMDMs and Tregs were cultured as mentioned in (A-C) but without DH treatment. CD4^+^ T cells from the MLN of naïve 6-8-week-old C57BL/6 mice were cultured in 96-well plate at 2×10^5^ cells/well and stimulated with anti-CD3 (1 μg/ml) plus anti-CD28 (1 μg/ml). For all four types of cells, total cell number was counted at 24 h, 48 h and 72 h after cell culture. Asterisks indicate the significance of the difference between CD4^+^ T cells and BMDCs (the second fastest growing cell type). **(E)** BMDCs and BMDMs were treated with LPS (1 μg/ml) in the absence (0 mg/L, DMSO control) or presence of different concentrations of DH as indicated. After 24 h, the concentration of IL-1β and IL-6 in the culture supernatant was determined by ELISA. **(F)** CD4^+^CD25^+^YFP^+^ Tregs were treated with anti-CD3 (1 μg/ml) plus anti-CD28 (1 μg/ml) in the absence (0 mg/L, DMSO control) or presence of different concentrations of DH as indicated. After 48 h, the concentration of IL-10 in the culture supernatant was determined by ELISA. For all panels, data are shown as the mean±SD of triplicates and are representative of two independent experiments. BMDCs: bone marrow-derived dendritic cells; BMDMs, bone marrow-derived macrophages; Tregs, regulatory T cells. MLN, mesenteric lymph node; n.s., no significance. **P < 0.01.

In addition to CD4^+^ T cells, we also evaluated the effect of DH on suppressing the proliferation of three other types of immune cells. Bone marrow-derived dendritic cells (BMDCs), bone marrow-derived macrophages (BMDMs) and regulatory T cells (Tregs) were treated with or without DH and cell number was counted after 48 h. We found that the number of BMDCs (**Figure 6A**), BMDMs (**Figure 6B**), and Tregs (**Figure 6C**) showed no significant difference in DH-treated and DMSO control group. To investigate why DH suppressed the proliferation of CD4^+^ T cells but not BMDCs, BMDMs and Tregs, we compared the growth curve of these four types of cells. Our results showed that the growth rate of BMDCs, BMDMs and Tregs is much less than that of CD4^+^ T cells (**Figure 6D**). We then reasoned that the effect of DH on suppressing cell proliferation is probably in proportion to the rate of cell growth. Consistent with these results, the production of IL-1β and IL-6 by BMDCs and BMDMs (**Figure 6E**) and IL-10 by Tregs (**Figure 6F**) was comparable between DH-treated and DMSO control group.

Taken together, these results demonstrate that DH is a potent agent to suppress conventional CD4^+^ T-cell proliferation and this suppression is in proportion to the rate of cell growth.

### DH-Treated CD4^+^ T Cells Are Partially Arrested at the G2/M Phase of the Cell Cycle

**Figure 7:**
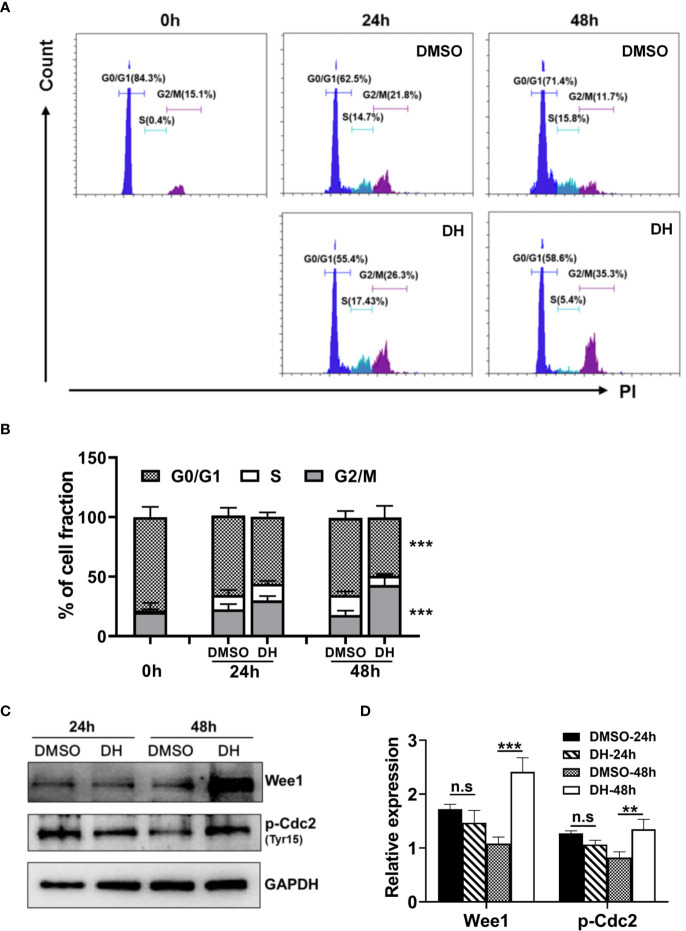
DH-treated CD4^+^ T cells are partially arrested at the G2/M phase of the cell cycle. **(A, B)** CD4^+^ T cells from the mesenteric lymph node (MLN) of naïve 6-8-week-old C57BL/6 mice were cultured in 96-well plate at 2×10^5^ cells/well and stimulated with anti-CD3 (1 μg/ml) plus anti-CD28 (1 μg/ml) in the absence (0 mg/L, DMSO control) or presence of DH (40 mg/L). Cells neither stimulated nor treated with DH were used as control (0 h). After 24 h or 48 h, cells were collected and stained with propidium iodide (PI) and the DNA contents were assayed by flow cytometry analysis. The histogram **(A)** and quantification **(B)** of the percentages of CD4^+^ T cells in the G0/G1, S, G2/M phase of the cell cycle were shown. **(C, D)** CD4^+^ T cells from the MLN of naïve 6-8-week-old C57BL/6 mice were cultured in 24-well plate at 5×10^6^ cells/well and treated as mentioned in **(A, B)**. The expression of p-cdc2 (Tyr15) and wee1 kinase was determined by western blotting at 24 h and 48 h. GAPDH expression was used as loading control. Data are representative **(A, D)** or shown as the mean±SD **(B, D)** of two independent experiments. n.s, no significance. ***P < 0.001.

To investigate which specific cell cycle phase was affected upon DH treatment, we examined the cell cycle phase distribution of proliferating CD4^+^ T cells by PI staining followed by flow cytometry analysis. Our results showed that DH-treated CD4^+^ T cells displayed a small but not significant reduction in cell-cycle progression at 24 h after stimulation. However, the reduction became apparent at 48 h, as evident by a marked increase in G2/M-phase cells and a concomitant decrease in G0/G1-phase cells (**Figures 7A, B**). To investigate why DH-treated CD4^+^ T cells were partially arrested at the G2/M phase of the cell cycle, we checked the phosphorylation of Cdc2 kinase, which is a key regulator of the transition from G2 phase to M phase. Our results showed that DH significantly increased the inhibitory phosphorylation (Tyr15) of Cdc2 at 48 h (**Figures 7C, D**). In addition, we also found that the expression of Wee1 kinase, a key negative regulator of Cdc2 kinase activity, was increased at 48 h after treatment with DH (**Figures 7C, D**). The increase of Wee1 kinase and the inhibitory phosphorylation (Tyr15) of Cdc2 was not obvious at 24 h. This is in consistency with the results from cell-cycle analysis, which showed no significant reduction in cell-cycle progression at that time-point. Collectively, these data indicate that DH promotes the inhibitory phosphorylation (Tyr15) of Cdc2, possibly through the promotion of Wee1 kinase expression, and consequently caused partial cell cycle arrest at G2/M phase.

### Treatment With DH Ameliorates EAU in Mice

**Figure 8:**
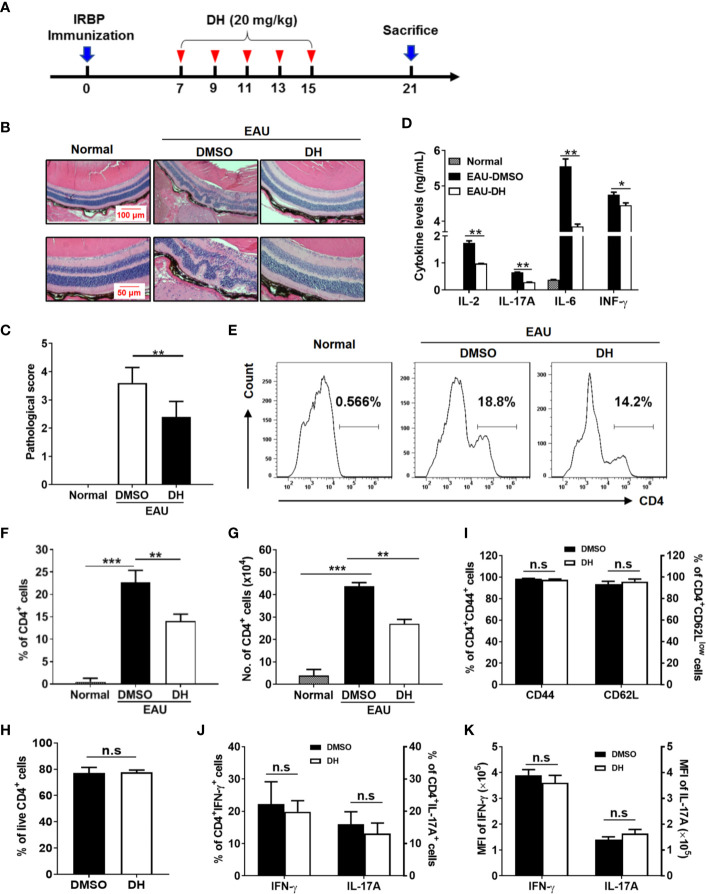
Treatment with DH ameliorates EAU in mice. **(A)** Schematic illustration of the induction and treatment of IRBP-induced EAU in mice. The red arrowheads indicate the treatment with DH or DMSO. The numbers indicate the day after IRBP immunization. **(B, C)** Mice were sacrificed at the 21st day after immunization and histopathological profiles of the eye were determined by hematoxylin and eosin staining. Normal mice, which neither induced EAU nor treated with DMSO or DH, were used as negative control. Ordered retina layers were shown in **(B)**. The severity of EAU (n=10) was evaluated in a masked fashion on a scale of 0-4 as described in the materials & methods **(C)**. **(D)** Mice were sacrificed at the 21st day after immunization and total cells were isolated from the eye and treated with anti-CD3 (1 μg/ml) plus anti-CD28 (1 μg/ml) for 48 h. The concentration of IL-2, IL-17A, IL-6 and IFN-γ in the culture supernatant was determined by ELISA. **(E–K)** Mice were treated as in **(A)** and sacrificed at the 21st day after immunization. Total cells were isolated from the eye, stained with FITC-anti-CD4, and analyzed by flow cytometry. The histogram **(E)** and quantification of the percentage **(F)** and absolute number **(G)** of CD4^+^ T cells were shown. Cells shown in histogram plots were gated on total live cells. To examine the degree of cell death, cells were stained first with FITC-anti-CD4 and then APC-anti-Annexin V and PI. The percentage of live CD4^+^ T cells (CD4^+^Annexin-V^−^PI^−^) within gated CD4^+^ T cells was determined by flow cytometry **(H)**. To examine CD4^+^ T cell activation, cells were stained with FITC-anti-CD4 plus APC-anti-CD44 or APC-anti-CD62L, and analyzed by flow cytometry **(I)**. To examine the production of inflammatory cytokines by CD4^+^ T cells using flow cytometry, cells were re-stimulated with PMA plus ionomycin in the presence of protein transport inhibitor for 4 h. Cell surface staining was first performed with FITC-anti-CD4. Cells were then fixed, permeated, and stained with PE-anti-IFN-γ and APC-anti-IL-17A. The percentages of CD4^+^IFN-γ^+^ and CD4^+^IL-17A^+^ cells **(J)** and the mean fluorescence intensity (MFI) of IFN-γ and IL-17A **(K)** were determined by flow cytometry analysis (gated on CD4^+^ T cells). Data shown are mean±SD **(C, F–K)** or representative **(B, D, E)** of two independent experiments. n.s, no significance. *P < 0.05, **P < 0.01, ***P < 0.001.

To investigate whether DH can be used to treat CD4^+^ T-cell–mediated inflammatory diseases, an IRBP-induced EAU mouse model was used. EAU was induced in C57BL/6 mice as described in the materials and methods and then mice were systemically administrated with equal volume of DMSO or 20 mg DH per kg body weight on day 7, 9, 11, 13 and 15 after IRBP immunization (**Figure 8A**). Normal mice, which neither induced EAU nor treated with DMSO or DH, were used as negative control. At day 21, mice were sacrificed and pathological changes in the retina were examined by H&E staining. As shown in **Figure 8B**, DH-treated mice exhibited much less retinal edema, structural distortion and inflammatory cell infiltration. When pathological changes in the retina were evaluated according to the scoring criteria previously reported, DH-treated mice had a mean score of 2.1, whereas that number for DMSO-treated mice is 3.6 (**Figure 8C**). Consistent with these results, the production of inflammatory cytokines including IL-2, IL-17A, IL-6, and IFN-γ from DH-treated mice was significantly decreased (**Figure 8D**).

### CD4^+^ T Cell Number Is Reduced in the Eye of DH-Treated Mice

**Figure 9:**
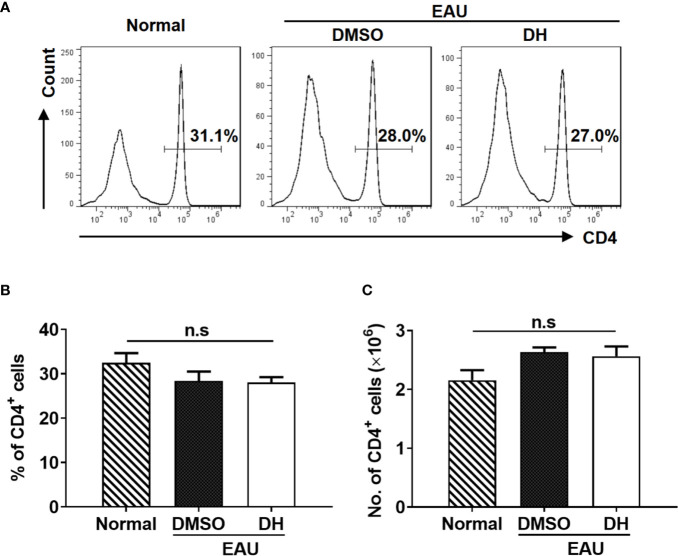
DH-treated mice exhibited normal percentage and number of CD4^+^ T cells in the cervical lymph node (CLN). Mice were treated as in **Figure 8** and sacrificed at the 21st day after immunization. Total cells were isolated from the cervical lymph node (CLN), stained with FITC-anti-CD4, and analyzed by flow cytometry. The histogram **(A)** and quantification **(B)** of the percentage of CD4^+^ T cells and the absolute number of CD4^+^ T cells **(C)** were shown. Cells shown in histogram plots were gated on total live cells. Data are representative **(A)** or shown as mean±SD **(B, C)** of two independent experiments. n.s, no significance.

We next examined the percentage and number of CD4^+^ T cells in the eye and CLN from normal, DMSO and DH-treated mice. The results showed that, while the percentage (**Figures 8E, F**) and absolute number (**Figure 8G**) of CD4^+^ T cells in the eye of IRBP-immunized mice were increased when comparing with normal mice, they were significantly decreased in DH treated mice when comparing with DMSO treated mice (**Figures 8E–G**). To exclude the possibility that DH affects CD4^+^ T cell death *in vivo*, we examined the percentage of live CD4^+^ T cells in the eye. Our results showed that the percentage of live CD4^+^ T cells in the eye showed no significant difference in DMSO and DH-treated mice (**Figure 8H**). In addition, there is no significant difference in the activation of CD4^+^ T cells (**Figure 8I**) and the percentage (**Figure 8J**) and the mean fluorescence intensity (MFI) (**Figure 8K**) of IFN-γ^+^ and IL-17A^+^ cells in DMSO and DH-treated mice. However, we found that the percentage (**Figures 9A, B**) and number (**Figure 9C**) of CD4^+^ T cells in the CLN were comparable among normal, DMSO and DH-treated mice.

**Figure 10:**
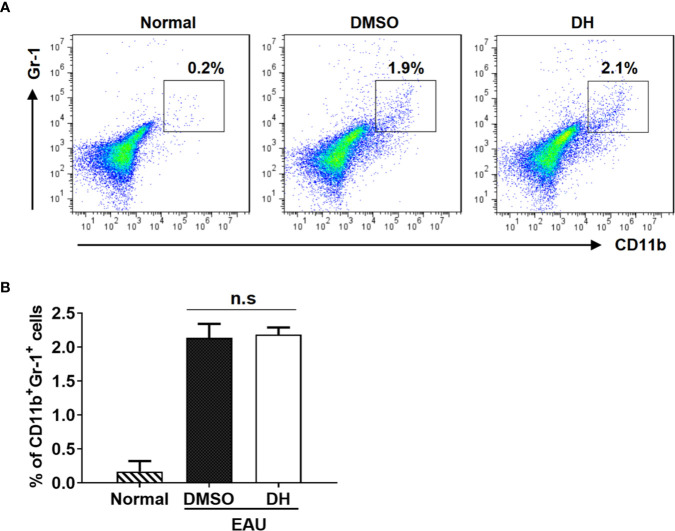
MDSCs recruitment in the eye was not affected in DH-treated mice. Mice were treated as in **Figure 8** and sacrificed at the 21st day after immunization. Cells were isolated from the eye and stained with FITC-anti-Gr1 and APC-anti-CD11b. The percentage of CD11b^+^Gr1^+^ cells was determined by flow cytometry. The dot plot **(A)** and quantification **(B)** of the percentage of CD11b^+^Gr1^+^ cells were shown. Cells shown in dot plots were gated on total live cells. Data are representative **(A)** or shown as mean±SD **(B)** of two independent experiments. MDSCs: myeloid-derived suppressor cells. n.s, no significance.

Previous study showed that DH suppressed the production of inflammatory cytokines by T cells through the recruitment of MDSCs to liver in conA-induced acute hepatitis model. To determine the potential effects of DH treatment on recruiting MDSCs in EAU model, we also examined the percentage of MDSCs in the eye. Our results showed that, although the percentage of CD11b^+^ Gr-1^+^ MDSCs was increased when EAU was induced, there was no significant difference of it between DMSO and DH treated mice (**Figures 10A, B**).

### DH Suppresses the Proliferation of Autoreactive Antigen-Specific CD4^+^ T Cells

**Figure 11:**
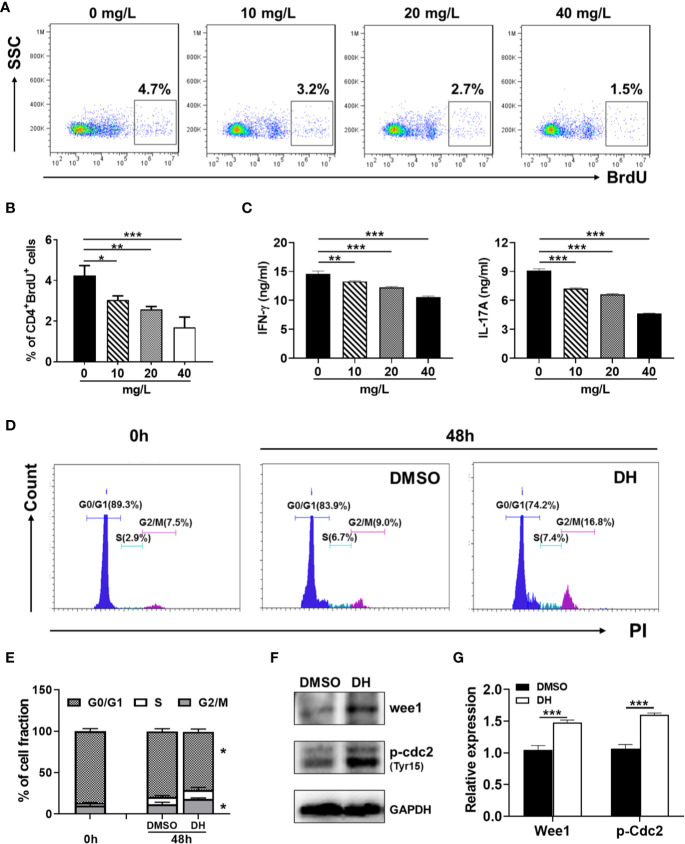
DH suppresses the proliferation of autoreactive antigen-specific CD4^+^ T cells. **(A–C)** CD4^+^ T cells were isolated from the CLN of IRBP-immunized mice 10 days after immunization. BMDCs were prepared from naïve C57BL/6 mice and treated with LPS (100 ng/ml) for 24 h. Matured BMDCs were furthered purified using EasySep™ Mouse CD11c Positive Selection Kit. 3 × 10^5^ CD4^+^ T cells were mixed with 0.6×10^5^ matured BMDC in 96-well plate and stimulated with IRBP peptide (30 μg/ml) in the absence (0 mg/L, DMSO control) or presence of DH (10, 20, and 40 mg/L). For the detection of cell proliferation, 10 μM BrdU was also added to the cell culture medium. After 72 h, cell surface staining was first performed with FITC-anti-CD4. Cells were then stained with APC-anti-BrdU and cell proliferation was analyzed by flow cytometry. The dot plot **(A)** and quantification **(B)** of the percentage of CD4^+^BrdU^+^ cells were shown. Cells shown in dot plots were gated on CD4^+^ T cells. Gating strategy for BrdU was based on control sample not stained with APC-anti-BrdU. In addition, the concentration of IFN-γ and IL-17A in the cell culture supernatant was determined by ELISA after 48 h **(C)**. **(D–F)** CD4^+^ T cells from the CLN of IRBP-immunized mice were stimulated as mentioned in **(A-C)** with the exception that only 40 mg/L of DH was used. Cells neither stimulated nor treated with DH were used as control (0 h). After 48 h, cells were stained with FITC-anti-CD4 and propidium iodide (PI) and DNA contents were assayed by flow cytometry analysis. The histogram **(D)** and quantification **(E)** of the percentages of CD4^+^ T cells in the G0/G1, S, G2/M phase of the cell cycle were shown. Cells shown in histogram plots were gated on CD4^+^ T cells. In addition, the expression of p-Cdc2 (Tyr15) and Wee1 kinase was determined by western blotting at 48 h **(F, G)**. GAPDH expression was used as loading control. Data shown are mean±SD **(B, E, G)** or representative (all other panels) of two independent experiments. CLN, cervical lymph node. *P < 0.05, **P < 0.01, ***P < 0.001.

To determine whether DH affects the proliferation of autoreactive antigen-specific CD4^+^ T cells, CD4^+^ T cells from the CLN of IRBP-immunized mice (10 days after immunization) were stimulated *in vitro* with IRBP-loaded BMDCs and treated with or without DH. When cell proliferation was assessed using an *in vitro* BrdU assay, we found that DH significantly suppressed the proliferation of IRBP-specific CD4^+^ T cells (**Figures 11A, B**). Consistent with this result, the production of IFN-γ and IL-17A by IRBP-specific CD4^+^ T cells was significantly decreased upon DH treatment (**Figure 11C**).

To determine whether DH treatment also leads to increased duration of the G2/M phase in autoreactive antigen-specific CD4^+^ T cells, we examined the cell cycle phase distribution of proliferating IRBP-specific CD4^+^ T cells by PI staining followed by flow cytometry analysis. Our results showed that DH-treated IRBP-specific CD4^+^ T cells displayed marked increase in G2/M-phase cells and a concomitant decrease in G0/G1-phase cells (**Figures 11D, E**). Accordingly, the expression of Wee1 kinase and inhibitory phosphorylation (Tyr15) of Cdc2 was significantly increased upon DH treatment (**Figures 11F, G**). Collectively, these data indicate that, in addition to suppress the proliferation of polyclonally activated naive CD4^+^ T cells, DH is also capable of suppressing antigen-induced proliferation of autoreactive CD4^+^ T cells.

## Discussion

*Dracocephalum heterophyllum* (DH) is a Chinese herbal medicine and has been used in treating liver diseases for decades. Studies have shown that DH extract significantly suppressed the production of inflammatory cytokines in ConA-induced hepatitis through recruiting MDSCs to the liver. Using EAU mouse model, our current study revealed a novel mechanism of DH-mediated suppression of T-cell function, i.e., suppressing CD4^+^ T-cell proliferation.

Traditional Chinese medicine has been used for treating inflammatory disease for thousands of years. It contains multiple components with diversities in chemical structures and biological activities, and each component exerts therapeutic effects in combination rather than as individuals. In the current study, although we have demonstrated that DH ethyl acetate extract can effectively suppress CD4^+^ T cell proliferation and ameliorate EAU in mice, we did not identify the active component in this extract. HPLC analysis revealed that this extract is composed of six main components. Most of them have the anti-oxidation, anti-inflammatory, antibacterial, and antitumor effects (23–29). Interestingly, three of them (methyl Rosmarinate, Luteolin and Diosmetin) are capable of suppressing cell proliferation. It has been reported that Diosmetin treated cells were arrested at the G1 phase of the cell cycle. Since DH treated CD4^+^ T cells were arrested at the G2/M phase, it is unlikely that DH suppressed CD4^+^ T cell proliferation through Diosmetin. Whether Rosmarinate and Luteolin are the active components in DH that caused CD4^+^ T cell proliferation arrest at the G2/M phase remains to be further explored. Nevertheless, the overall effects of DH on CD4^+^ T cell proliferation may be the result of synergistic effect of all components that are capable of suppressing cell proliferation.

After differentiation and development in the thymus, CD4^+^ T cells are distributed to the immune organs and tissues of the whole body through lymphatic and blood circulation. As an important component of the immune system, CD4^+^ T cells play an indispensable role in resisting foreign pathogens and mediate the development of autoimmunity (30, 31). Abnormal CD4^+^ T-cell proliferation has been implicated in the development of various diseases including peripheral T-cell proliferative diseases/lymphomas, inflammatory/infection diseases, graft-versus-host disease and autoimmune diseases (13, 32, 33). Regulatory T cells (Tregs), as well as transforming growth factor-β (TGF-β) and IL-10, are known to have important inhibitory functions in CD4^+^ T-cell proliferation (34–36). In addition, a variety of plants have been implicated to have the ability to regulate immune response (37, 38). Indeed, our current study revealed that DH exhibits potent anti-proliferative effects on autoreactive CD4^+^ T cells and ameliorates the development of experimental autoimmune uveitis.

The transition from G2 phase to M phase of the cell cycle is mainly regulated by CDK1 kinase, also known as Cdc2 or M-phase promoting factor (MPF). Coupled with cyclinB1, the phosphorylation and dephosphorylation of this protein plays important regulatory roles in G2/M phase transition of eukaryotic cell cycle. Our results showed that the inhibitory phosphorylation (Tyr15) of Cdc2 was increased upon DH treatment. This indicates that DH likely caused partial cell cycle arrest at G2/M phase through the inhibition of Cdc2 activity. Previous study has shown that Wee1 kinase is a key negative regulator of Cdc2 activity. Indeed, our results showed that the expression of Wee1 kinase was increased upon DH treatment. However, since Cdc2 activity is regulated through multiple mechanisms during the cell cycle progression, we can’t rule out the possibility that DH regulates Cdc2 activity through additional mechanism(s). In addition, DH may inhibit CD4^+^ T cell proliferation through mechanisms other than promoting the inhibitory phosphorylation of Cdc2 (Tyr15). Finally, as we mentioned before, it remains to be further explored to identify the active components in the DH extracts that can promote the inhibitory phosphorylation of Cdc2 (Tyr15).

Although an increasing number of studies have proven the significance of DH in the treatment of various human diseases, the underlying mechanism of action is still elusive. To obtain an effective strategy for disease treatment, the interactions of natural molecules with cellular targets should be clarified. It has been reported that DH extract significantly suppressed the production of inflammatory cytokines in ConA-induced hepatitis through recruiting MDSCs to the liver (16). However, we didn’t observe altered MDSC recruitment in DH-treated mice when using EAU mouse model. Although CD4^+^ T cells play important roles in both ConA-induced hepatitis (39) and EAU, apparently treatment with DH suppressed the production of inflammatory cytokines by CD4^+^ T cells through different mechanisms in these two disease models. Though it remains to be determined whether DH also suppressed CD4^+^ T-cell proliferation in ConA-induced hepatitis model, the disparity regarding the mechanism of DH in suppressing cytokine production by CD4^+^ T cells reveals that DH may play distinct roles under different disease conditions.

CD4^+^ T cells are quite complex and heterogeneous, and CD4^+^ T-cell subsets with different developmental stages or functions can exist at the same time. Tregs are a special subset of CD4^+^ T cells that prevent autoimmunity and inflammation. Defects in Tregs cause severe inflammatory disease (40, 41). If DH also suppresses the proliferation of Tregs, by doing so it may actually accelerate the development of EAU. Our results showed that the effect of DH on suppressing the proliferation of Tregs is much less than that on conventional CD4^+^ T cells, probably because the growth rate of Tregs is much less than that of conventional CD4^+^ T cells. This hypothesis is further supported by the fact that DH didn’t suppress the proliferation of BMDCs and BMDMs, two important immune cell types whose growth rate is also much less than that of conventional CD4^+^ T cells. Indeed, we also found that DH exhibits anti-proliferative effects on fast growing CD8^+^ T cells (data not shown). Therefore, we reasoned that DH ameliorates EAU in mice primarily through the inhibition of the proliferation of conventional CD4^+^ T cells.

During the development of IRBP-induced EAU in mice, IRBP-specific CD4^+^ T cells encountered antigen, activated and undergone expansion in the cervical lymph nodes (CLN). They then leave CLN and infiltrate into the retina and cause inflammatory lesion (41, 42). Although we have shown that the percentage and number of CD4^+^ T cells were greatly decreased in the eye of DH-treated mice, the difference in the percentage and number of CD4^+^ T cells in the CLN was not evident. We think this is because only a small number of cells in the CLN can respond to antigen stimulation. Decreased number of CD4^+^ T cells in the eye upon DH treatment may reflect that the number of antigen-specific CD4^+^ T cells is decreased in the CLN.

Uncontrolled cell proliferation is the hallmark of cancer, and tumor cells have typically acquired damage to genes that directly regulate their cell cycles (43, 44). Our current study showed that DH has relative stronger inhibitory effect on fast-growing cells, which indicates that it may also regulate the proliferation of tumor cells. Indeed, our preliminary data (data not shown) revealed that DH significantly suppressed the proliferation of A875 cells (a human melanoma cell line) *in vitro*. Interestingly, this result is in consistency with published data which showed that three main components of DH (methyl Rosmarinate, Luteolin and Diosmetin) are able to inhibit tumor cell proliferation. Further study is needed to clarify the role of DH in regulating tumor cell proliferation and whether it can suppress tumor growth *in vivo*.

In summary, our current study revealed a novel mechanism of DH-mediated suppression of CD4^+^ T-cell function. Since DH has already been widely used in treating human diseases, our current research provides theoretical basis for the new application of DH in treating diseases that are associated with abnormal CD4^+^ T-cell proliferation.

## Data Availability Statement

All datasets presented in this study are included in the article/ supplementary material.

## Ethics Statement

The animal study was reviewed and approved by Shandong Eye Institute, P. R. China.

## Author Contributions

QR designed research. JB, KW, and TW performed research. QW and PW contributed reagents/analytic tools. JB, WS, and QR analyzed data. JB and QR wrote the paper. All authors contributed to the article and approved the submitted version.

## Funding

This work was supported by the National Natural Science Foundation of China (81530027); Shenzhen Science and Technology Innovation Commission (JCYJ20170413165432016, JCYJ20180507182525623); The Major New Drug Creation Special Projects (2019zx09201001); The International Cooperation Project of Qinghai Province (2020-HZ-804).

## Conflict of Interest

The authors declare that the research was conducted in the absence of any commercial or financial relationships that could be construed as a potential conflict of interest.
